# The immunosuppressive face of sepsis early on intensive care unit—A large-scale microarray meta-analysis

**DOI:** 10.1371/journal.pone.0198555

**Published:** 2018-06-19

**Authors:** Dominik Schaack, Benedikt Hermann Siegler, Sandra Tamulyte, Markus Alexander Weigand, Florian Uhle

**Affiliations:** Department of Anesthesiology, Heidelberg University Hospital, Im Neuenheimer Feld 110, Heidelberg, Germany; South Texas Veterans Health Care System, UNITED STATES

## Abstract

**Background:**

Sepsis is defined as a life-threatening condition, resulting from a dysregulated and harmful response of the hosts’ immune system to infection. Apart from this, the (over-)compensating mechanisms counterbalancing the inflammatory response have been proven to render the host susceptible to further infections and increase delayed mortality. Our study aimed to unravel the heterogeneity of immune response in early sepsis and to explain the biology behind it.

**Methods:**

A systematic search of public repositories yielded 949 microarray samples from patients with sepsis of different infectious origin and early after clinical manifestation. These were merged into a meta-expression set, and after applying sequential conservative bioinformatics filtering, an in-deep analysis of transcriptional heterogeneity, as well as a comparison to samples of healthy controls was performed.

**Results:**

We can identify two distinct clusters of patients (cluster 1: 655 subjects, cluster 2: 294 subjects) according to their global blood transcriptome. While both clusters exhibit only moderate differences in direct comparison, a comparison of both clusters individually to healthy controls yielded strong expression changes of genes involved in immune responses. Both comparisons found similar regulated genes, with a stronger dysregulation occurring in the larger patient cluster and implicating a loss of monocyte and T cell function, co-occurring with an activation of neutrophil granulocytes.

**Conclusion:**

We propose a consistent—but in its extent varying—presence of immunosuppression, occurring as early in sepsis as its clinical manifestation and irrespective of the infectious origin. While certain cell types possess contradictory activation states, our finding underlines the urgent need for an early host-directed therapy of sepsis side-by-side with antibiotics.

## Introduction

Critically ill patients on intensive care units (ICU) are affected by complex and frequently interwoven medical conditions. Above others, sepsis continues to challenge worldwide modern intensive care medicine as the main contributor to mortality and critical illness on ICU.[1,2]

Recently, the syndrome sepsis has been defined as a dysregulated host response to infection associated with a life-threatening organ dysfunction.[3] While organ dysfunction is a new aspect, the combination of infection with a dysregulated immune reaction of the host is a constant throughout the development of clinical definition. [4,5] Besides this, early attempts have been undertaken to frame the central immune response into a simplified pathophysiological concept. After leaving the incomplete concept of sepsis as a sole pro-inflammatory syndrome, a dynamic shift from an initial pro-inflammatory response, named systemic inflammatory response syndrome (SIRS), to a delayed and counteracting compensatory anti-inflammatory response syndrome (CARS) was hypothesized.[6] Due to the co-occurrence of pro- and anti-inflammatory mediators in the blood of patients with sepsis, the idea of a sequential staging of the syndrome has been abolished and replaced by a model anticipating that both SIRS and CARS occur at the same time in a so-called mixed antagonist response syndrome (MARS). However, the concept does not consider pathophysiological subtypes of the syndrome resulting from the individual predisposition of the host as well as the attributes of the microbiological insult.[7] As a consequence, more than hundred clinical trials targeting pro-inflammatory processes in a “one-size-fits-all” approach failed [8,9], apparently pointing towards the urgent need for a better understanding of the complex and interwoven pathophysiology.[10]

Several studies approached the pathophysiological complexity of sepsis by measuring the meta-transcriptome of circulating immune cells using microarrays. Predominantly, the results further foster the idea of sepsis as a continuum of states rather than a binary condition merely switching between pro- and anti-inflammation.[11] Nevertheless, the studies largely varied in their design, especially regarding patient characteristics, time of sampling and analyzed cell types, impeding a direct comparison and extraction of a universal conclusion.

In our study, we investigate the early whole blood transcriptome of adult patients with sepsis irrespective of the underlying focus of infection. In total, we merged 949 individual microarray samples from patients with sepsis available from public repositories into a meta-expression set to facilitate data-level analysis of heterogeneity of the group of patients. Based on the global gene expression, we found two distinct clusters of sepsis patients and comparing them to samples of healthy subjects drives evidence for highly divergent states of immune cell activation as early as sepsis is clinically apparent.

## Methods

### Microarray data selection

**Fig 1:**
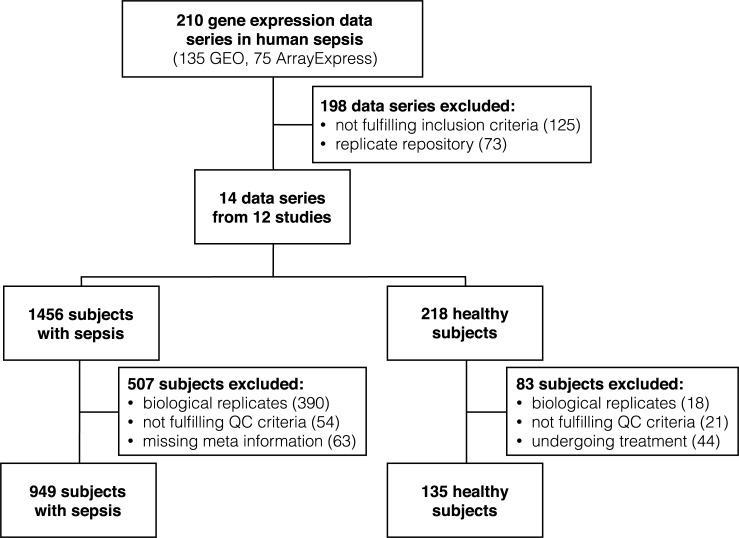
Flowchart of microarray data selection. Data series collected from GEO and ArrayExpress were subjected to a selection process resulting in 14 data series from 12 studies. Samples of patients with sepsis and healthy controls were further assessed to meet various standards for analysis.

We screened two public repositories for global gene expression data series (NCBI Gene Expression Omnibus (GEO, National Centre for Biotechnology Information) and EMBL-EBI ArrayExpress). Using the search term “sepsis [AND] homo sapiens”, we retrieved 135 GEO- and 75 ArrayExpress results (date of retrieval: 12/31/2016). Three researchers independently reviewed these results concerning in- and exclusion criteria. Included data series had to be obtained (i) via microarray-technique from (ii) whole blood samples of (iii) adult patients with (iv) sepsis that has been diagnosed based on international consensus definitions (v) immediately after admission to ICU. Samples from patients with sepsis triggered by a viral infection (e.g. H1N1 influenza) were excluded. Also, replicate results in both screened repositories were excluded, leading to a collection of raw microarray expression data from ten studies selected from the GEO database (GSE prefix). Two additional studies, each including two discrete data series were obtained from ArrayExpress archive (E-MTAB prefix) (S1 Table). The resulting 14 data series included 1456 patients with sepsis and 218 healthy subjects. After excluding technical replicates, subjects not fulfilling QC criteria (see supplementary methods S1 File for details) and healthy controls undergoing treatment, the final numbers decreased to 949 septic patients and 135 healthy controls (Fig 1 and Table 1). The source repository, unique ID as well as analysis platform used for the initial studies are given in S1 Table.

**Table 1:** Overview of included studies with indication of available metadata and number of samples (both retrievable and published). BS: Bloodstream, CAP: community-acquired pneumonia, FP: fecal peritonitis.

			Metadata		Published samples		Retrievable samples	
First author	Year	Focus	Mortality	Age	Sepsis	Healthy	Sepsis	Healthy
Pankla [12]	2009	BS	-	+	31	8	13	5
Howrylak [13]	2009	Various	-	-	42	0	34	0
Sutherland [14]	2011	Various	-	-	10	20	10	20
Dolinay [15]	2012	Various	-	-	122	0	48	0
Parnell [16]	2013	Various	+	+	35	18	35	0
Ahn [17]	2013	BS	-	+	51	43	51	43
Cazalis [18]	2014	Various	-	+	28	25	28	25
McHugh [19]	2015	Various	-	-	74	0	74	0
Scicluna [20]	2015	CAP	-	+	171	0	108	42
Kangelaris [21]	2015	Various	-	+	57	0	57	0
Davenport [22]	2016	CAP	+	+	384	0	367	0
Burnham [23]	2017	CAP, FP	+	+	335	0	124	0

### Bioinformatics and statistical analyses

The detailed analysis pipeline and workflow is given in the supplementary material (S1 File). The meta-datasets used in this study are freely available to download from the Synapse repository (www.synapse.org) under the project ID doi:10.7303/syn11932743.

## Results

### Molecular heterogeneity in early sepsis

**Fig 2:**
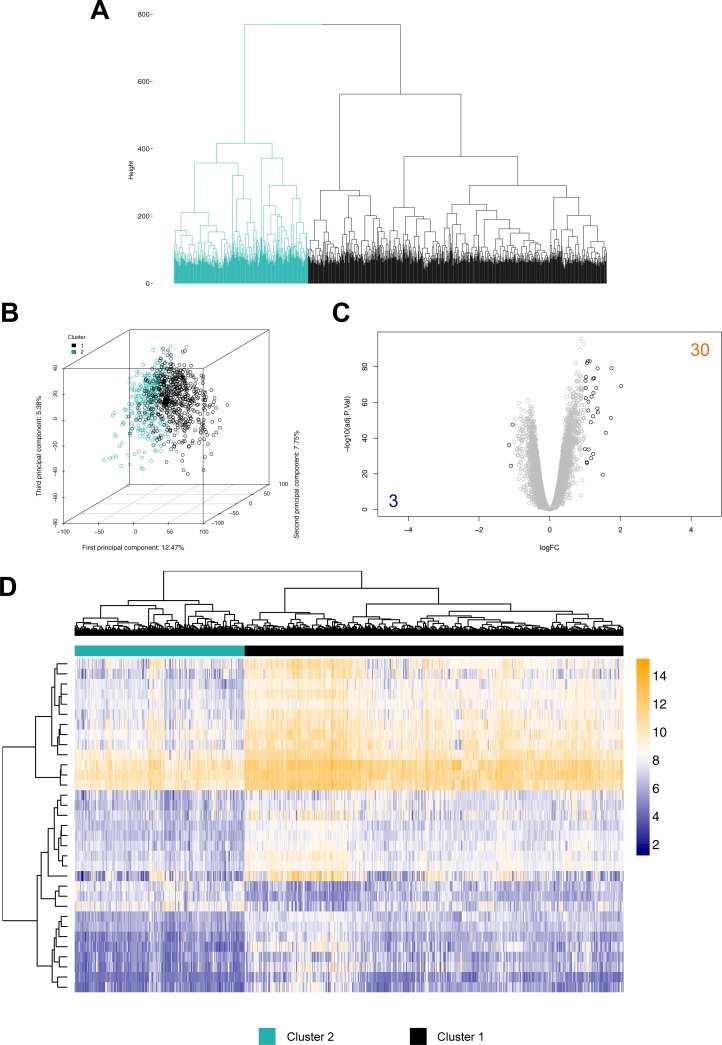
Hierarchical cluster analysis of microarray expression data provided for the 5,000 most-variable gene symbols in 949 patients of the septic group. (A) Dendrogram illustrating the arrangement of the clusters produced: Cluster 1 (gray) comprises 655 subjects, while cluster 2 (cyan) includes 294 individuals. (B) Scatterplot showing the amount of data variance explained by the first three principal components. Subjects are colored according to their respective cluster assignment. (C) Volcano plot showing the gene symbols differentially expressed (solid black color highlights results with absolute logFC ≥ 1, adjusted *p*-value < 0.05). Resulting number of significant genes above the defined absolute logFC threshold are indicated by numbers (orange: up-regulated, blue: down-regulated). (D) Heatmap depicting processed expression values of the 33 differentially regulated genes for all cluster-assigned individuals.

By applying hierarchical clustering to the remaining 949 sepsis samples (containing the 5,000 most variable genes), we can readily identify two distinct transcriptional signatures (Fig 2A and 2B): 655 samples grouped into Cluster 1 (highlighted in gray), while the remaining 294 samples grouped into Cluster 2 (highlighted in cyan). The latter exhibits lower intra-cluster heterogeneity compared to Cluster 1, observable by the larger distance to first branching (Fig 2A). The majority of included studies fed samples into both clusters; thereby a systematic bias introduced by inter-study effects can be precluded (S2 Table). Also, cluster affiliation of samples based on their appropriate microarray chip type can be ruled out (S3 Table). Comparison of gene expression between the clusters revealed 33 genes to be differentially regulated (absolute log_2_FC ≥ 1, adjusted *p*-value < 0.05), with 30 up- and 3 down-regulated genes (Fig 2C and 2D, Table 2, S2 File). A subsequent analysis of implicated biological processes revealed no significant overrepresentation of a certain process. Nevertheless, when approaching the differentially regulated genes by network analysis, the activation of several cell signaling pathways seems reasonable (S5 Fig). Genes belonging to the Interleukin receptor family (e.g. *IL18R*, *IL18RAP*, *IL1R2*), as well as acute phase response genes (e.g. haptoglobin (*HP*) and its corresponding scavenger receptor *CD163*), are dysregulated amongst others (for full list, see S2 File). Moreover, genes indicative of metabolic differences are also up-regulated in Cluster 1, e.g. *ARG1* and *HDPG*.

**Table 2:** Top five up- and down-regulated genes according to the different comparisons. Log_2_FC: Log_2_ fold change; adj.: adjusted; *HPGD*: 15-hydroxyprostaglandin dehydrogenase; *IL18R1*: Interleukin-18 Receptor I; *OLAH*: Oleoyl-ACP Hydrolase; *CD*: Cluster of Differentiation; *MMP8*: Matrix metalloproteinase 8; *GZMH*: Granzyme H; *GZMK*: Granzyme K; *HP*: Haptoglobin; *RETN*: Resistin; *GNLY*: Granulysin; *IL7R*: Interleukin-7 Receptor; *IL2RB*: Interleukin 2 Receptor Subunit Beta; *HLA-DRB1*: Human Leucocyte Antigen DRB1; *IFI27*: Interferon Alpha Inducible Protein 27; *CHI3L1*: Chitinase 3-Like 1; *TRBC1*: T Cell Receptor Beta Constant 1; *CCR3*: C-C Motif Chemokine Receptor 3; *TRDV2*: T Cell Receptor Delta Variable 2.

C1 vs. C2			C1 vs. Healthy			C2 vs. Healthy			
Gene	Log_2_FC	adj. *p*-value	Gene	Log_2_FC	adj. *p*-value	Gene	Log_2_FC	adj. *p*-value	
*HPGD*	2,02	7,1E^-70^	*CD177*	3,86	3,9E^-127^	*CD177*	2,27	2,7E^-44^	↑
*IL18R1*	1,75	7,4E^-80^	*MMP8*	3,49	7,7E^-60^	*MMP8*	1,99	2,8E^-18^	
*OLAH*	1,73	4,9E^-52^	*HP*	3,16	2,3E^-104^	*HP*	1,99	1,2E^-39^	
*CD177*	1,58	1,0E^-45^	*RETN*	2,78	2,0E^-76^	*IFI27*	1,96	2,4E^-18^	
*MMP8*	1,50	2,8E^-20^	*OLAH*	2,72	2,8E^-77^	*RETN*	1,85	3,1E^-31^	
			*GNLY*	-1,91	3,9E^-65^	*CHI3L1*	-1,35	3,6E^-19^	↓
			*IL7R*	-1,94	1,5E^-76^	*TRBC1*	-1,44	1,0E^-168^	
*CD27*	-1,05	2,5E^-48^	*CD27*	-1,96	4,0E^-93^	*HLA-DRB1*	-1,45	8,1E^-21^	
*GZMH*	-1,10	3,0E^-25^	*IL2RB*	-1,98	7,6E^-101^	*CCR3*	-1,49	2,8E^-53^	
*GZMK*	-1,14	8,1E^-37^	*HLA-DRB1*	-2,12	5,5E^-50^	*TRDV2*	-1,52	4,8E^-167^	

In summary, despite a clear distinction is possible between the clusters based on their global gene expression, only a small number of genes are pointing out in direct comparison.

### The biological identity of the clusters

To extract functional differences of the two clusters, which might have been blurred by the initial inter-cluster comparison, we conducted in the next step a comparison of each cluster individually to the samples of healthy controls (n = 135).

**Fig 3:**
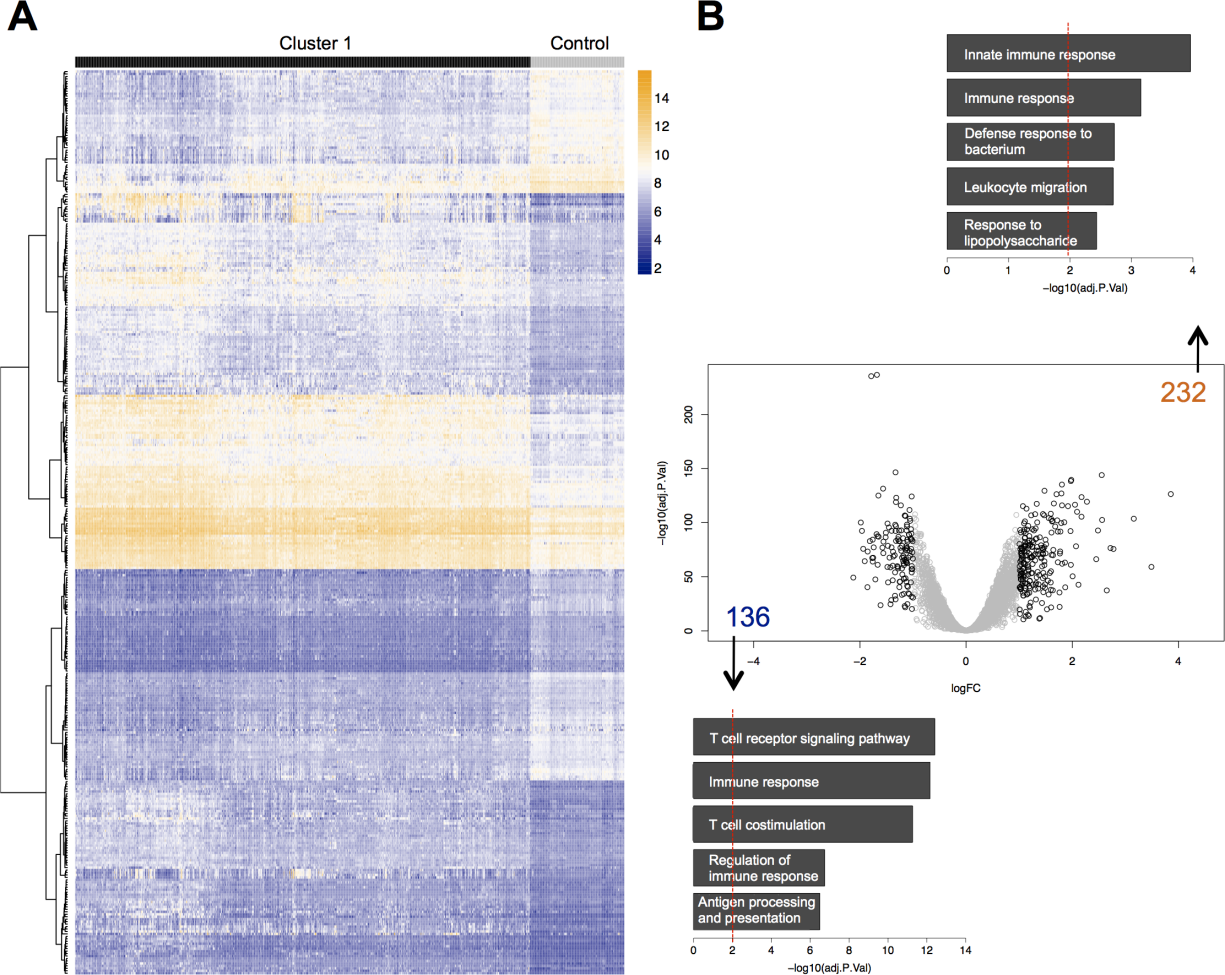
Differential expression analysis between “Cluster 1” and individuals from the healthy control group. (A) Heatmap of processed expression values for 368 dysregulated genes showing absolute logFC ≥ 1 (adj. *p*-value < 0.05). (B) Volcano plot of differentially expressed genes (solid black color indicates absolute logFC ≥ 1.0, adj. *p*-value < 0.05; numbers indicate up- (orange) or down-regulated (blue) genes). Results of GO-term analysis for enriched biological processes separately for up-regulated (top panel) and down-regulated genes (bottom panel) above defined threshold.

Cluster 1 was found to exhibit 368 differentially regulated genes, with 232 up- and 136 down-regulated genes (logFC ≥ 1, adj. *p*-value < 0.05) (Fig 3, S2 Table, S3 Table). GO term analyses proved many genes belonging to immune system-related biological functions to be regulated in both directions, with “innate immune response” (GO:0002226) being the top up-regulated function and “T cell receptor signaling pathway” (GO:0050852) the top down-regulated one. Both T-cells as well as antigen-presenting cells seem to experience an early loss of core function, with reduced expression of a variety of genes necessary for T cell receptor signaling or co-stimulation (e.g. *CD3D*, *CD3E*, *CD3G*, *TRBC1*, *CD8A*, *CD247*) respectively antigen presentation (e.g. *CD74*, *HLA*-*DMA/-DMB/-DPA1/-DPB1/-DRB1/-DRB3/-DRB5*). Surprisingly, the classical pro-inflammatory cytokines often postulated as important drivers of sepsis, e.g. IL-6, are not regulated in any manner. The only cytokine genes found regulated include the calgranulin *S100A12*, anti-inflammatory *IL10*, and inflammasome-dependent *IL18*. To facilitate the processing of the last one, two integral components of the inflammasome, *AIM2* and *CASP5* are also up-regulated. In addition, the observed up-regulation of (counter-) receptors of interleukin-1 and -18 (IL1R2, IL18R, IL18RAP), still holds true when comparing patients with sepsis to healthy controls. Nevertheless, as not less than 4 of the 5 most up-regulated genes (*CD177*, *MMP8*, *HP*, *RETN*) in the Cluster 1 signature (as well as Cluster 2 signature) can be allocated to activated neutrophils, a pro-inflammatory activation of these cells seems obvious (Table 2, S3 File).

**Fig 4:**
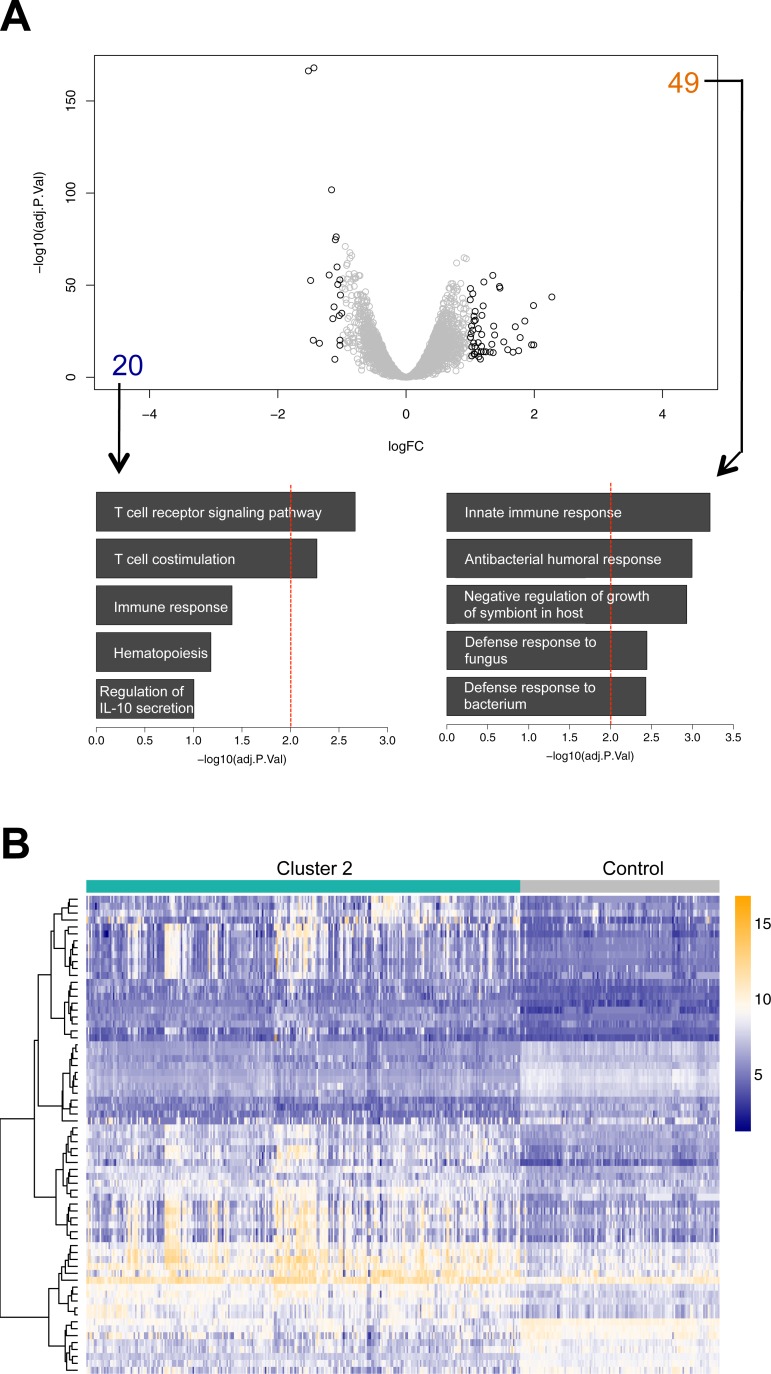
Differential expression analysis between “Cluster 2” and individuals from the healthy control group. (A) Volcano plot of differentially expressed genes (solid black color indicates absolute logFC ≥ 1.0, adj. *p*-value < 0.05; numbers indicate up- (orange) or down-regulated (blue) genes) and results of GO-term analysis for enriched biological processes separately for down-regulated (left panel) and up-regulated genes (right panel) above defined threshold (B).

**Fig 5:**
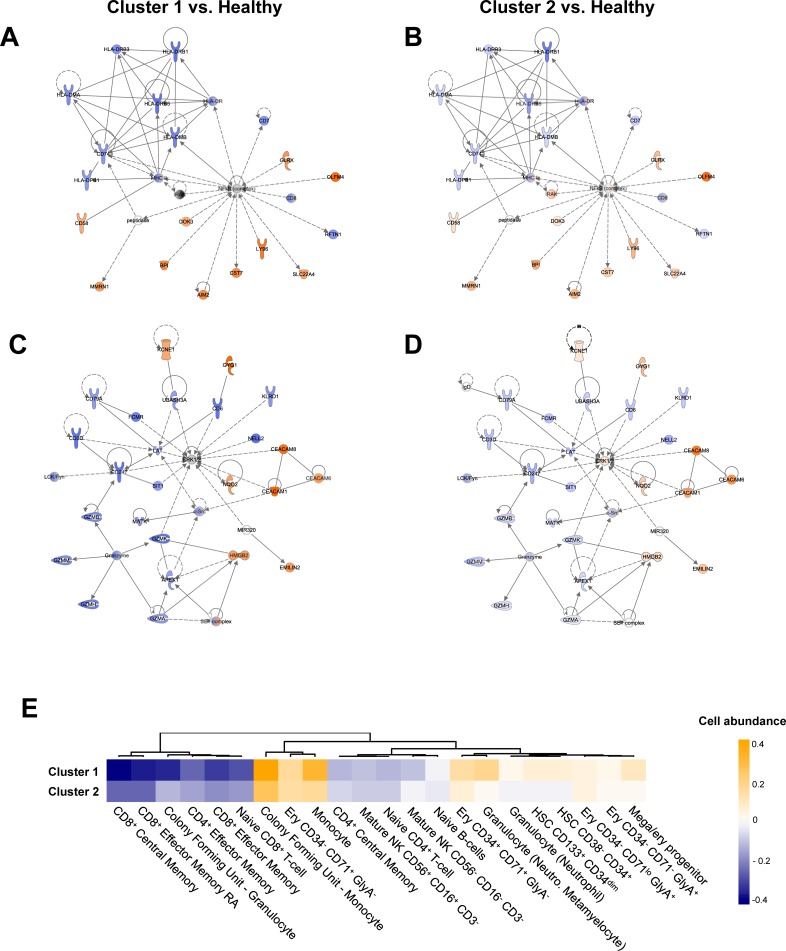
Networks of differentially expressed genes between defined clusters and healthy controls. (A) Top network of differentially regulated genes between patients of “Cluster 1” and healthy individuals. (B) Manually selected network consisting of differentially regulated immune-related genes. Nodes showing an orange color implicate up-regulation for the conditions in contrast, while blue elements represent down-regulation. (C) and D) Overlays of the respective expression data for “Cluster 2” subjects in comparison to individuals from control group. (E) Heatmap showing the results of data deconvolution to identify cell origin of signals. Orange color represents an up-regulated “cell abundance”, representing more signals deriving from this cell type compared to healthy controls, blue color vice versa. Only informative cell types were visualized. Mega: megakaryocyte; Ery: erythroid; HSC: hematopoetic stem cell.

A comparison of samples belonging to Cluster 2 with healthy controls revealed a similar result regarding functional implications with a total of 69 genes found differentially regulated (49 up- and 20 down-regulated genes) (Fig 4, S4 File). In fact, with the exception of 6 genes exclusively up-regulated in Cluster 2, there is a consensual dysregulation of genes in both clusters with a more pronounced up- or down-regulation in Cluster 1 patients, also observable in a graphical representation of the top networks (Fig 5A–5D, S5 Fig).

**Fig 6:**
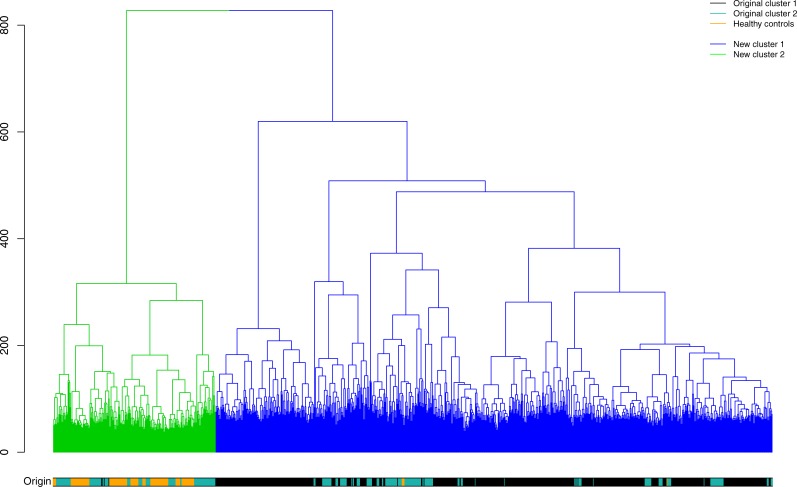
Hierarchical cluster analysis of microarray expression data provided for the 5,000 most-variable gene symbols in the full dataset of 1084 subjects. Dendrogram and color track (origin) illustrate the sample re-arrangement of the clusters produced: New cluster 1 (blue) consists of 839 subjects, while 245 individuals are attributed to cluster 2 (green). In comparison to Fig 2A, new cluster 1 identity is unchanged in most cases (99.4% retention rate), while adding new subjects from original cluster 2. The 245 individuals assigned to new cluster 2 cover both original cluster 2 samples as well as the vast majority of healthy controls (96.3% assignment rate).

To verify these results the combined dataset consisting of 949 septic subjects and 135 healthy controls was re-clustered. 839 subjects are assigned to new cluster 1 (Fig 6; highlighted in blue) and the residual 245 samples to new cluster 2 (highlighted in green). When comparing sample identity of original cluster 1 and new cluster 1, 651 of 655 samples (99.4%) retain their original cluster identity, while 4 samples (0.6%) are now re-assigned to new cluster 2. Original cluster 2 holds 294 samples. 111 of these subjects (37.8%) are still attributed to new cluster 2, while 183 samples (62.2%) are now belonging to new cluster 1. The majority of controls 130 of 135 (96.3%) are assigned to new cluster 2, while just 5 samples (3.7%) are matched to new cluster 1. This result supports the original sample assignments (Fig 2A). Furthermore, a systematic bias in regard to sample origin by relationship to originating study (S4 Table) or microarray chip type does not emerge (S5 Table).

To identify the cell types, which contribute to the observed gene expression, we conducted a data deconvolution using the exploratory ImmQuant tool [24]. The results further supported our gene expression analysis, with a pronunciation of signals deriving from monocytes and granulocytes together with a robust underrepresentation of all T cell subsets (Fig 5E). Interestingly, our analysis also provides evidence that immature immune cells are involved in the early response, including e.g. granulocyte and monocyte progenitors besides hematopoietic stem cells.

In summary, our findings provide evidence for a quantitative difference of gene expression levels between the clusters rather than a qualitative contrast based on distinctly regulated gene sets. Moreover, activation states of different immune cells might vary.

### Extraction of gene sets for diagnosis and cluster stratification

Ten differentially regulated genes (*IL1R2*, *CD177*, *HPGD*, *MMP8*, *HP*, *ARG1*, *OLFM4*, *HLA-DRB1*, *IL7R*, *AZU1*) were manually selected according to their degree of expressional dysregulation compared to controls, functional context, as well as the potential cell of signal origin. The biological rationale was to cover the main immune cell types proposed to be involved in sepsis pathophysiology, including T cells, monocytes, and granulocytes.

Expression levels of single genes exhibit already a high diagnostic AUC, with *CD177* (0·955), *HP* (0·935) and *IL7R* (0·921) being the most prominent ones (S6 Fig). Before further analysis, the dataset was randomly split into a derivation and a validation set (60:40). Binary logistic regression for sepsis (both clusters) vs. healthy controls extracted a diagnostic panel of five genes (*CD177*, *HLA-DRB1*, *HP*, *IL7R*, *AZU*1) and subsequent ROC analysis of combined probability revealed an AUC of 0·977 (CI: 0·967–0·987; derivation set) respectively 0.985 (CI: 0.974–0.996; validation set) (S7A Fig). Using the same approach for cluster stratification, we found six genes (*IL1R2*, *HPGD*, *ARG1*, *HP*, *IL7R*, *AZU1*) enabling cluster stratification with an AUC of 0·893 (0·866–0·920; derivation set) respectively 0.897 (CI: 0.865–0.930; validation set) (S7B Fig). Altogether, we can extract a panel of eight genes (three of them shared for the approaches) capable to distinguish between the sepsis clusters as well as between sepsis and healthy controls.

## Discussion

Since the birth of microarray technology around the end of the last millennium, an enormous number of microarray experiments have been performed on uncountable cell types, conditions, and diseases. Earlier (meta-)analysis of sepsis microarrays were often performed in a narrative approach or by the re-analysis of individual studies. Contrasting, our study systematically searched, extracted and merged samples of 949 patients with sepsis and generated to our knowledge the largest meta-expression set available.

We identified two distinct clusters, whose expression mainly differed in the quantitative dimension of gene expression rather than in the actual genes regulated. Comparing the clusters to a group of healthy samples, we were able to further underline the qualitative similarity of both clusters, placing patients from Cluster 2 on an intermediate state between healthy and Cluster 1 patients. The samples included in our study were derived from 12 studies performed with different intentions; the majority of studies aimed to identify biomarker signatures for diagnostic purpose. McHugh and colleagues generated the 4-gene SeptiCyte Lab classifier to discriminate between sepsis and sterile inflammation. [14,19] By first comparing the groups of patients with and without CAP to healthy controls and secondly the results against each other, Scicluna et al. yielded similar dysregulated genes as our study did (e.g. *MMP8*, *CD177*, and *HP*).[20] Especially the gene encoding for the acute phase protein haptoglobin is standing out as well in our study with the signal most likely derived from activated granulocytes. Haptoglobin has been shown earlier to be of potential usefulness for the diagnosis of sepsis in preterm and term newborns.[25] Importantly, in our results *HP*, as well as the corresponding receptor for haptoglobin-hemoglobin-complexes *CD163*, are positively co-regulated, implicating an important role of iron homeostasis in early sepsis. Haptoglobin has been proposed as an anti-inflammatory compound acting on immune cells in a receptor-mediated manner, and through scavenging of free hemoglobin thereby reducing oxidative stress and draining substrate from pathogens. [26,27] Recently, the receptor CD163 has been shown to exert anti-inflammatory effects after binding to *High Mobility Group Box 1* (HMGB1) protein-hemoglobin complexes.[28] HMGB1 itself is a well-known damage-associated molecular pattern (DAMP), also binding to *Toll-like* receptor 4 (TLR4) and the *Receptor for Advanced Glycation Endproducts* (RAGE) and is under debate as a therapeutic target.[29]

The study conducted by Howrylak et al. aimed to identify genes associated with acute respiratory distress syndrome (ARDS) and therefore compared septic patients with and without ARDS.[13] They found an eight-gene panel able to discriminate between both states. A second study conducted years after the initial study and using the same design with an extended number of patients found different dysregulated genes, most likely deriving from activated neutrophils, with *MMP8*, *OLFM4*, and *HP* again belonging to the most up-regulated ones.[21] Deriving from our meta-analysis, we are able to identify a novel 5-gene diagnostic classifier as well as a 6-gene cluster classifier, both with high sensitivity and specificity. However, prospective validation using a PCR-based methodology has yet to be performed. Interestingly, three of the five diagnostic genes are cell surface markers (CD177, HLA-DRB1, IL7R) of neutrophils, monocytes and T cells, respectively, raising the potential for rapid assessment by flow cytometry. The *HLA-DRB1* gene encodes for the beta chain of the heterodimeric *Major Histocompatibility Complex* (MHC) Class II, responsible for antigen-presentation on monocytes and B cells. Reduced abundance of MHC-II (as found in our results) has been extensively proven as a valid surrogate marker of (innate) immune dysfunction in sepsis and trauma.[30] Contradictory, CD177 is a surface marker of neutrophil activation and has been discovered as the most dysregulated marker in isolated neutrophils of septic patients.[31] Together with the prospective results of our data deconvolution, this hints towards the hallmark of emergency myelopoiesis and immature progenitor cells in the circulation taking place in early sepsis.[32,33] The receptor for IL-7 is expressed in T cells and reduced expression indicating an “exhausted” phenotype has been shown earlier in prolonged and fatal sepsis.[34,35]

In a comparable meta-approach like ours, the group of Khatri used samples of patients with trauma or SIRS in comparison to patients with sepsis to extract both a diagnostic gene signature (the 11-gene Sepsis MetaScore [36]) as well as an additional 7-gene set to discriminate viral from bacterial infections.[37] Remarkably, although they used a fundamentally different control group compared to our analysis, several genes were comparably changed. This finding questions the often-proposed rationale of using patients with a sterile SIRS as a comparator to distill the proportion of host response attributable to infection. Especially the idea of generally using patients after surgery per se as patients with sterile SIRS is faulty, as only a minor fraction of these patients clinically impose with a SIRS. Interestingly, the group utilized samples from adult as well as pediatric patients in both studies. The immune system, especially of neonates, is immature in several aspects with e.g. a not fully developed adaptive branch regarding memory B and T cells.[38,39] Given that, we did not use such samples in our analysis to reduce heterogeneity by study design.

Dolinay et al. focused on inflammasome-regulated cytokines during ARDS and found an up-regulation of the inflammasome component *ASC* as well as *IL1B* gene, the latter together with IL-18 also found in the plasma of enrolled patients.[15] In our analysis, we can confirm the robust up-regulation of several genes belonging to the IL1/18 axis, e.g. the decoy receptor IL1R2 together with IL18R. IL1-R2 is counteracting inflammation by binding IL-1beta without intracellular signaling transduction, and its gene expression has been reported earlier to be of high diagnostic usefulness for sepsis, also after trauma.[40,41] Similar results have been shown for inflammatory IL-18 and blocking it together with IL-1β protected animals against inflammation and shock.[42]

Two further studies enrolled small numbers of patients with sepsis respectively septic shock and found genes associated with disturbed immune function already early in the course of the disease. [16,18] Both studies compared patients with sepsis to healthy controls; Cazalis et al. found no correlation of the gene signature with SAPSII score, while Parnell et al. found a pronounced dysregulation of identified genes in non-surviving patients compared to survivors. In line with our findings, pathways involving T cell receptor and CD28 signaling as well as antigen presentations where massively down-regulated already early after sepsis onset. In a large-scale prospective cohort, Davenport et al. evaluated the heterogeneity of CAP sepsis and identified two transcriptome signatures, with the smaller one being associated with immunosuppressive alterations and a reduced 14-day survival, most likely due to the occurrence of secondary infections.[22] While their cluster comparison yielded a large number of differentially regulated genes, this was not the case with our results. We observed only subtle changes in gene expression between our clusters as well as among patients in Cluster 2 and healthy controls, leading us to the conclusion that patients in our “Cluster 2” possess a transitory signature half the way to full immunosuppression. In a second study, the group examined patients with abdominal sepsis due to a fecal peritonitis and found again two signatures with different mortality and gene expression.[23] Remarkably, when comparing patients with fecal peritonitis to CAP patients, the genomic signatures showed large overlaps. As no comparison of the two clusters to healthy subjects has been performed in both studies, the absolute biological function of each clusters seems illusive and it is unclear, if immunosuppression as proposed in the initial study is not also present in the cluster used as a comparator, yet in a lower extent.

Our study has some limitations, not least based on the use of foreign data. First, due to the nature of a meta-analysis and the algorithms used to conduct the analysis, our study should be anticipated as exploratory and hypothesis-generating rather than confirmatory. Second, clinical meta-data of patients included in our analysis is only sparsely available, hampering an in-deep correlation of observed signatures with clinical characteristics. Third, by applying the strict filter steps of our pipeline, we might have excluded informative genes due to a lack of representation in all studies, respectively samples. Last, with the lack of hematological data, an observed reduction or increase in gene expression might be either based on changes in cell abundance or the actual change in transcription. Nevertheless, the resulting implication remains the same: a loss or gain of function in the compartment blood based on the derived signal.

**Fig 7:**
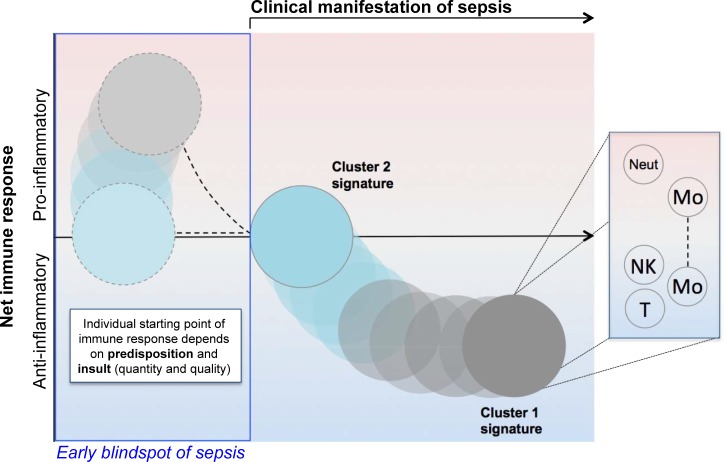
Pathophysiological model of sepsis genomic response. The early blindspot of sepsis (blue box) spans the highly individual timeframe from infection to clinical manifestation of symptoms. The quantitative and qualitative kinetic of response depends on both host and pathogen attributes. Our results originating from samples of patients early after ICU admission for sepsis prove the presence of (at least) two molecular signatures of sepsis (Cluster 1 and Cluster 2), with Cluster 1 implicating a higher degree of dysregulation towards immunosuppression than Cluster 2. Within the clusters, different cell types are likely to have contradictory or even ambivalent activation states, e.g. monocytes (Mo) with impaired cytokine production but with maintained migratory function. Neut: neutrophilic granulocytes; NK: natural killer cells; T: T cells.

The concept of early sepsis as a solely pro-inflammatory syndrome should be finally discarded and replaced by a multi-dimensional concept, taking into consideration different cell types, their opposing polarization and activation states as well as the ideas of cellular immune memory and tolerance (Fig 7). When sepsis becomes clinically apparent, the initial reaction of the immune system has been mounted hours ago and might already be counter-regulated in certain cell types, rendering them refractory to further stimuli. This concept has been well described for monocytes, which enter a state of “endotoxin tolerance” upon an initial stimulus by e.g. LPS, mediated and sustained by epigenetic mechanisms.[43,44] On the other hand, cellular functions can be regulated independent of each other, and a loss of cytokine production must not necessarily run along a decreased phagocytic activity. The “sepsis blindspot” of treatable pro-inflammation occurs early and outside the clinical scope, but its immunological echo can be misleadingly measured at later times due to the half-time of plasma cytokines. Therefore, the window to take clinical action in terms of anti-inflammatory therapies is gone. Extrapolating from our data we generate a model of early and robust counter-regulation of immune cell activation as early as sepsis is clinically apparent. The signals can be allocated to T cells, monocytes and neutrophils, with a transient post-activation gene signature (“Cluster 2”), which drifts further into deep immunosuppression (“Cluster 1”). Overall, there is a rising need for a system’s approach for biomarker-guided and personalized adjunctive therapy of sepsis, moving away from the estimate of a system direction towards a cell-type individual response. Although removed from the recent definition for clinical diagnosis, sepsis stays an immunopathology with a central hub of a systemic response to infection taking place in the blood compartment, rendering it a highly important claim for pathophysiological data-mining in the future.

## Conclusion

We need to reframe our perspectives on sepsis syndrome: Highly individual predisposition and the qualitative and quantitative characteristic of the insult mount a response, which can be “late sepsis” from start or “early sepsis” for days, with divergent activation states of different immune cells. Based on this concept, we need to identify the pathological phenotypes of the cells and the corresponding host-directed therapies. Several of which might be already available from oncology, but if they project into a reduction of antibiotic use, survival and quality of life of the patients has yet to be proven.

## Supporting information

S1 Fig Flowchart of microarray data (pre-)processing (1.) and annotation to official human gene symbols (2.). The process of expression set combination was repeated until the final meta-expression set was created (3.). Data from septic patients (n = 949) were separated from the complete expression set of 1,084 individuals for further analysis (4.).
(EPS)

S2 Fig Schema of combination of the meta-expression set. Generic Biobase expression set objects are based on a single matrix of processed expression values which is extended by two attributing metadata matrices containing feature and phenotype data. To combine two expression sets with differing identifiers, expression values of fully comparable identifiers are merged (step 1). Data relating to identifiers unique to the first expression set is appended, while respective data fields reserved for the second expression set are left unchanged (step 2). The former process is repeated to include identifiers and data values exclusively present to the second expression set (step 3). Feature data matrices are combined with the same strategy as described for expression data. Phenotype data from both expression sets are concatenated.
(EPS)

S3 Fig Flowchart of the meta-expression set filtering, imputation and normalization strategy. Top workflow: The meta-expression set is duplicated. Expression data for septic patients is separated from the full dataset. The matrix of expression values is limited to gene symbols available in at least four originating data series. Missing expression values per gene symbol are imputed based on expression data of the 10 closest subjects with sepsis. Expression values are normalized between cohorts. To reduce the number of non-informative genes, requirements regarding minimal expression values for single gene symbols are applied for further consideration. The top-5000 candidates of the remaining gene set are selected for subsequent unsupervised hierarchical cluster analysis. Lower workflow: To prepare healthy control data, expression values in the full dataset are replaced by imputation. Data for subjects of the sepsis and control sub-groups were processed separately. Batch effect adjustment is performed on the full meta-expression set, normalizing healthy controls against septic patient data. To enable comparison between clusters and controls, the top-5000 genes as defined for patients with sepsis are selected from control subjects included in the full meta-expression set.
(EPS)

S4 Fig Analysis of differentially expressed genes unified from comparisons between both clusters against healthy controls. A) Heatmap of calculated logFC values for the respective contrasts. B+C) Venn diagrams distinctively highlighting the total number of dys-regulated genes shared or unique by both clusters according to their direction of regulation.
(EPS)

S5 Fig Top network generated from an IPA analysis of the 33 differentially regulated genes between the two clusters. Nodes showing an orange color implicate up-regulation of gene in “Cluster 1”, while blue elements represent down-regulation. Grey nodes depict predicted active pathways contributing to observed gene expression.
(EPS)

S6 Fig Receiver-operator-characteristic curve for the 10 individual genes selected for logistic model generation regarding diagnostic performance (A). The corresponding areas under curve are given in panel B. Values below 0·5 result from genes down-regulated in patients with sepsis.
(EPS)

S7 Fig Receiver-operator-characteristic curve plots for the identified gene panels to discriminate patients with sepsis from healthy controls (A) or patients with sepsis of the two clusters (B). Upper plots depict results from model derivation analysis (60% of samples), bottom plots the results from validation using the remaining samples (40%). Corresponding sample size used for each group and analysis is given in the plot heading. Number in plot gives Area-under-curve and corresponding confidence interval (in brackets). Genes included in the underlying signature are given between the ROC curves.
(EPS)

S1 Table Overview of included studies with indication of microarray platform used for data generation and repository ID of data series. (DOCX)

S2 Table Distribution of sepsis samples by data series ID among clusters. (DOCX)

S3 Table Distribution of sepsis samples by platform type among clusters. (DOCX)

S4 Table Distribution of combined samples by data series ID among clusters. (DOCX)

S5 Table Distribution of combined samples by platform type among clusters. (DOCX)

S1 File Supplementary methods. (DOC)

S2 File Results of „Cluster 1”vs. „Cluster 2”comparison of the 5000 most variable genes. (XLS)

S3 File Results of „Cluster 1”vs. „healthy controls”comparison of the 5000 most variable genes. (XLS)

S4 File Results of „Cluster 2”vs „healthy controls”comparison of the 5000 most variable genes. (XLS)
