# Supplementary material for: The immunosuppressive face of sepsis early on intensive care unit—A large-scale microarray meta-analysis
Source: PLoS One. 2018 Jun 19;13(6):e0198555. doi: 10.1371/journal.pone.0198555 (PMC6007920; doi:10.1371/journal.pone.0198555)
Supplement: S1 Table — (DOCX) [file pone.0198555.s008.docx]

|  |  | **Microarray platform** | | |  |
| --- | --- | --- | --- | --- | --- |
| **First author** | **Year** | **Vendor** | **Identifier** | **Name** | **Repository ID** |
| Pankla | 2009 | Illumina | GPL6947 | HumanHT-12 V3.0 expression beadchip | GSE13015 |
| Howrylak | 2009 | Affymetrix | GPL571 | HG-U133A_2 | GSE10474 |
| Sutherland | 2011 | Affymetrix | GPL570 | HG-U133_Plus_2 | GSE28750 |
| Dolinay | 2012 | Illumina | GPL10558 | HumanHT-12 V4.0 expression beadchip | GSE32707 |
| Parnell | 2013 | Illumina | GPL6947 | HumanHT-12 V3.0 expression beadchip | GSE54514 |
| Ahn | 2013 | Affymetrix | GPL571 | HG-U133A_2 | GSE33341 |
| Cazalis | 2014 | Affymetrix | GPL570 | HG-U133_Plus_2 | GSE57065 |
| McHugh | 2015 | Affymetrix | GPL5175 | HuEx-1_0-st | GSE74224 |
| Scicluna | 2015 | Affymetrix | GPL13667 | HG-U219 | GSE65682 |
| Kangelaris | 2015 | Affymetrix | GPL6244 | HuGene-1_0-st | GSE66890 |
| Davenport | 2016 | Illumina | GPL10558 | HumanHT-12 V4.0 expression beadchip | E-MTAB-4421  E-MTAB-4451 |
| Burnham | 2017 | Illumina | GPL10558 | HumanHT-12 V4.0 expression beadchip | E-MTAB-5273  E-MTAB-5274 |
