# Supplementary material for: The immunosuppressive face of sepsis early on intensive care unit—A large-scale microarray meta-analysis
Source: PLoS One. 2018 Jun 19;13(6):e0198555. doi: 10.1371/journal.pone.0198555 (PMC6007920; doi:10.1371/journal.pone.0198555)
Supplement: S2 Table — (DOCX) [file pone.0198555.s009.docx]

| **First author** | **Data series ID** | **Cluster 1 (n=)** | **Cluster 2 (n=)** | **Ratio C1/C2** |
| --- | --- | --- | --- | --- |
| Pankla | GSE13015 | 9 | 4 | 2,25 |
| Howrylak | GSE10474 | 24 | 10 | 2,4 |
| Sutherland | GSE28750 | 8 | 2 | 4 |
| Dolinay | GSE32707 | 34 | 14 | 2,43 |
| Parnell | GSE54514 | 15 | 20 | 0,75 |
| Ahn | GSE33341 | 35 | 16 | 2,19 |
| Cazalis | GSE57065 | 19 | 9 | 2,11 |
| McHugh | GSE74224 | 46 | 28 | 1,64 |
| Scicluna | GSE65682 | 75 | 33 | 2,27 |
| Kangelaris | GSE66890 | 37 | 20 | 1,85 |
| Davenport | E-MTAB-4421 | 160 | 101 | 1,58 |
|  | E-MTAB-4451 | 69 | 37 | 1,86 |
| Burnham | E-MTAB-5273 | 71 | 0 | 0 |
|  | E-MTAB-5274 | 53 | 0 | 0 |
| **Total** |  | **655** | **294** | **2,23** |
