# Supplementary material for: The immunosuppressive face of sepsis early on intensive care unit—A large-scale microarray meta-analysis
Source: PLoS One. 2018 Jun 19;13(6):e0198555. doi: 10.1371/journal.pone.0198555 (PMC6007920; doi:10.1371/journal.pone.0198555)
Supplement: S3 Table — (DOCX) [file pone.0198555.s010.docx]

| **Vendor** | **Identifier** | **Name** | **Cluster 1 (n=)** | **Cluster 2 (n=)** | **Ratio C1/C2** |
| --- | --- | --- | --- | --- | --- |
| Illumina | GPL10558 | HumanHT-12 V4.0 expression beadchip | 387 | 152 | 2,55 |
| Affymetrix | GPL13667 | HG-U219 | 75 | 33 | 2,27 |
| Affymetrix | GPL5175 | HuEx-1_0-st | 46 | 28 | 1,64 |
| Affymetrix | GPL570 | HG-U133_Plus_2 | 27 | 11 | 2,46 |
| Affymetrix | GPL571 | HG-U133A_2 | 59 | 26 | 2,27 |
| Affymetrix | GPL6244 | HuGene-1_0-st | 37 | 20 | 1,85 |
| Illumina | GPL6947 | HumanHT-12 V3.0 expression beadchip | 24 | 24 | 1 |
| **Total** |  |  | **655** | **294** | **2,23** |
