# Supplementary material for: The immunosuppressive face of sepsis early on intensive care unit—A large-scale microarray meta-analysis
Source: PLoS One. 2018 Jun 19;13(6):e0198555. doi: 10.1371/journal.pone.0198555 (PMC6007920; doi:10.1371/journal.pone.0198555)
Supplement: S4 Table — (DOCX) [file pone.0198555.s011.docx]

| **First author** | **Data series ID** | **Cluster 1 (n=)** | **Cluster 2 (n=)** | **Ratio C1/C2** |
| --- | --- | --- | --- | --- |
| Pankla | GSE13015 | 13 | 5 | 2,6 |
| Howrylak | GSE10474 | 26 | 8 | 3,25 |
| Sutherland | GSE28750 | 10 | 20 | 0,5 |
| Dolinay | GSE32707 | 45 | 3 | 15 |
| Parnell | GSE54514 | 22 | 13 | 1,69 |
| Ahn | GSE33341 | 55 | 39 | 1,41 |
| Cazalis | GSE57065 | 29 | 24 | 1,21 |
| McHugh | GSE74224 | 66 | 8 | 8,25 |
| Scicluna | GSE65682 | 107 | 43 | 2,49 |
| Kangelaris | GSE66890 | 47 | 10 | 4,7 |
| Davenport | E-MTAB-4421 | 208 | 53 | 3,93 |
|  | E-MTAB-4451 | 87 | 19 | 4,58 |
| Burnham | E-MTAB-5273 | 71 | 0 | 0 |
|  | E-MTAB-5274 | 53 | 0 | 0 |
| **Total** |  | **839** | **245** | **3,43** |
