# Supplementary material for: The immunosuppressive face of sepsis early on intensive care unit—A large-scale microarray meta-analysis
Source: PLoS One. 2018 Jun 19;13(6):e0198555. doi: 10.1371/journal.pone.0198555 (PMC6007920; doi:10.1371/journal.pone.0198555)
Supplement: S5 Table — (DOCX) [file pone.0198555.s012.docx]

| **Platform type** | **Chip type** | **Cluster 1 (n=)** | **Cluster 2 (n=)** | **Ratio C1/C2** |
| --- | --- | --- | --- | --- |
| GPL10558 | Illumina HumanHT-12 V4.0 | 464 | 75 | 6,19 |
| GPL13667 | Affymetrix HG-U219 | 107 | 43 | 2,49 |
| GPL5175 | Affymetrix HuEx-1_0-st | 66 | 8 | 8,25 |
| GPL570 | Affymetrix HG-U133_Plus_2 | 39 | 44 | 0,89 |
| GPL571 | Affymetrix HG-U133A_2 | 81 | 47 | 1,72 |
| GPL6244 | Affymetrix HuGene-1_0-st | 47 | 10 | 4,7 |
| GPL6947 | Illumina HumanHT-12 V3.0 | 35 | 18 | 1,94 |
| **Total** |  | **839** | **245** | **3,43** |
