# Supplementary material for: The immunosuppressive face of sepsis early on intensive care unit—A large-scale microarray meta-analysis
Source: PLoS One. 2018 Jun 19;13(6):e0198555. doi: 10.1371/journal.pone.0198555 (PMC6007920; doi:10.1371/journal.pone.0198555)
Supplement: S1 File — (DOC) [file pone.0198555.s013.doc]

**The immunosuppressive face of early sepsis’ host response - insights from a large-scale meta-analysis of microarray data**

Dominik Schaack, M.Sc., Benedikt Hermann Siegler, M.D., Sandra Tamulyte, Markus Alexander Weigand, M.D., Florian Uhle, Ph.D.

**Supplementary methods**

*Processing and annotation*

Data formatting was carried out by in-house scripts to enable further processing in the R/Bioconductor environment.[1] Raw microarray expression data was first processed separately for all chosen data series to enable subsequent normalization between samples.[2] For the steps of reading-in the various expression matrices, RMA background correction and between array quantile normalization packages ***limma***, ***oligo*** and ***affy***[3,4], although comparable with regard to data import, had to be utilized in conjunction due to technical constraints. Where applicable, advanced background correction for selected Affymetrix platforms was provided by the ***gcrma*** package to make use of the probe sequence information for estimation of probe affinity to non-specific binding. To conclude data preparation steps, all expression values provided were log_2_-transformed.

Expression values of the 14 data series selected were measured on a total of seven divergent microarray platforms provided by Affymetrix and Illumina respectively. To facilitate comparison between individual samples, a solution was elaborated to enable mapping the diverse vendor- and platform-specific microarray probes to a common set of identifiers. The fundamental strategy pursued was based on the Bioconductor package ***crossmeta***: At first, all data series were annotated platform-wise using the information provided by the respective Bioconductor annotation packages. To be able to obtain a definite one-to-one mapping between microarray probes of all data series, the corresponding NCBI Entrez ID was extracted for all annotated probes. In case one microarray probe ID maps to different Entrez IDs, the corresponding expression values were multiplicated according to the number of matching Entrez IDs. In addition NCBI HomoloGene database was used to attribute the so-acquired Entrez IDs for all samples to official gene symbols for Human. In the first step mapping of Entrez IDs to Human Entrez IDs was conducted. The official gene symbol for each microarray probe was then derived from the genome wide annotation package for Human (***org.Hs.eg.db***) by utilizing the Annotation Database Interface (***AnnotationDbi***) together with ***annotationTools.***[5]

For each data series, analysis-ready expression set objects (derived from the ***Biobase*** package) were re-created out of the expression data prepared. Feature data and naming for the new expression sets were deduced from the corresponding annotation data and the arranged mapping to gene symbols respectively. Sample-specific phenotype data was either supplied by ***GEOquery*** for the studies selected from Gene Expression Omnibus database or by manual sample data import for chosen ArrayExpress data series[6].

*Phenotype data preparation and implementation of expression set combination*

Before constructing the final meta expression set, the respective study ID together with sample-specific group assignments for septic patients and subjects of the control group were appended individually to the phenotype data entries of all expression sets.

The process of combining preprepared expression set matrices was adapted from the data merging strategy outlined in the recently unsupported Bioconductor package ***inSilicoMerging.***[7] Its original methodology published was extended by two features critical to fit necessary requirements of the meta analysis: 1) To provide a stable solution for the one to one mapping of all gene symbols in the final meta expression set, a rational selection strategy for one expression value per gene for each combined expression set had to be implemented: If a gene symbol appears more than one time in a expression set (natively occurring due to redundant probes or as a result of the data multiplication described above), the expression value offering the highest interquartile range (IQR) measure is chosen out of multiple entries. 2) In comparison to other meta analysis strategies published[8]*,* ***inSilicoMerging*** source code was limited to exclusively merge the least common set of gene identifiers between different expression sets. Due to gene symbol feature set heterogeneity introduced by involving experiments facilitated on the basis of different microarray chips provided by multiple vendors, the original strategy of combination would have led to a significantly lower number of available gene symbols in the final meta expression set. Before adapting the source code to an integrative combining process, the resulting number of genes was less than 25% of the currently achieved output.

The combining process itself was re-designed and executed in a five-step iterative manner and repeated until all selected expression sets had been included in the final meta expression set: I) By comparison of two expression sets, the expression data values of all equivalent gene symbols are merged. II) Both expression data subsets for gene symbols exclusively occurring within one of both expression sets are concatenated to the newly merged expression set, while expression value data fields of the other sample are filled with blanks for these gene symbols. III) The according gene feature data matrices are merged and concatenated following the same strategy as for the processed expression data. IV) For phenotype data the sample-specific entries of both expression sets are appended to preserve all metadata available in both expression sets to be combined. V) A new expression set object is constructed out of the combined expression and feature matrices as well as the summarized phenotype data while fully maintaining matrix-subject-relationships.

The steps described above in conjunction with the changes to the original methodology of the ***inSilicoMerging*** package enable the stringent and unambiguous mapping of expression values and lossless concatenation of metadata required to implement a solution for combining two multi-dimensional data matrix objects like expression sets. The detailed mechanism of combining expression set objects is shown in supplemental Fig S1.

*Imputation, batch effect removal and quality control*Since the original samples originated from 14 different data series, a inter-series normalization of expression values between cohorts was vital before proceeding[2]. The meta expression matrix was prepared with the Bioconductor package ***impute*** to fill-in missing expression values based on the 10 nearest neighbors in gene space. Subsequently, ComBat (package: ***sva***) methodology was applied to the completed meta expression matrix to adjust for batch effects. The algorithm was supplied with metadata covering relationships of subjects to originating data series.

Missing values in the expression set duplicate containing the full dataset of septic patients and healthy controls were equally imputed. The process of imputation was applied separately for both groups of subjects. Normalization of batch effects was performed on the entire expression set to adjust expression values of the control group against septic patients.

For quality control the full expression set was assessed with principal component analysis (PCA). In addition to PCA-based QC assessment results confirmed by the authors of the originating data series which led to the exclusion of a total of 26 septic samples, 28 subjects with sepsis and 21 healthy control samples showing unadjustable batch effects were excluded from the final expression set. As depicted in supplementary Fig S2 following QC the numbers of subjects had been reduced to 949 septic samples and 135 healthy individuals as control group. After data exclusion steps for imputation and batch effect removal were repeated to reflect the changes to the full expression set comprising the reduced number of 1,084 subjects providing processed expression values for 28,544 unambiguous gene symbols together with all corresponding metadata for further downstream analyses.

*Filtering, minimum information content, variability and clustering*

The combined meta-expression set was duplicated. One copy was restricted to the group of septic patients, while the other copy was left unchanged conserving the complete set of subjects (Supplementary Fig S3). To define a final gene set of interest for patients with sepsis, the respective limited expression set was filtered to fulfill a minimal requirement of data content, while maintaining the advantages of the microarray meta analysis approach: Every gene symbol included in the resulting matrix had to be supported by at least 4 independent microarray data series. At the same time gene symbols indicating high amounts of missing expression data were discarded. This approach reduced the number of available gene symbols in the expression matrix of sepsis patients to 12,705.

To meet concern regarding microarray information content, an expression value of ≥ 5 in at least 10% of all subjects was selected to be the minimum required to retain a single gene symbol in the meta expression matrix of septic patients.[9] Furthermore, for the resulting 8,331 gene symbols a subset of the 5,000 most-variable candidates were identified to define a final gene set of interest for subsequent cluster analysis in the group of septic patients.[10] To enable between-group comparison, ComBat normalized expression data of healthy controls was subset for the defined gene set of interest.

The optimal number of clusters in the meta expression set of septic patients and the respective combined set of 1,084 samples was assessed by CRAN packages ***factoextra*** and ***FactoMineR*** on scaled expression data of the top-5,000 most variable gene symbols.[11] Based on a preliminary hierarchical clustering total within-group sum of square methodology identified for both sample compilations 2 as optimum value for further analyses.[12] Given the number of expected clusters, final clustering results were generated with Ward’s hierarchical agglomerative clustering method (Euclidean distance measure; Ward2 criterion).[13]

*Data availability*

All individual datasets used in this study are available from public repositories (for identifier, please see Supplementary table 1). The meta-datasets (all patients with sepsis, sepsis and healthy controls combined; total genes as well as top 5000 selection) used in this study are available to download from the Synapse repository (www.synapse.org) under the project ID doi:10.7303/syn11932743.

*Differential expression, GO-term and network analyses*

Results of differential expression analysis between defined clusters and the group of healthy controls were obtained by ***limma.***[14] The lowest absolute LogFC value between conditions required for considering results in GO-term and pathway analyses was defined as 1.0, while holding adjusted *p*-values below 0.05. Correction for multiple testing was carried out by the use of Benjamini-Hochberg procedure. For GO-term analysis the results were uploaded to the Database for Annotation, Visualization and Integrated Discovery (DAVID, https://david.ncifcrf.gov).[15] The network analyses were generated through the use of IPA (Ingenuity® Systems, www.ingenuity.com).

p

*Data deconvolution*

Hypothesis-generating ImmQuant software pipeline was used to apply deconvolution methodology for predicting differences in cell-type quantities between cluster-assigned subjects.[16] Microarray expression data for the unified subset of 374 gene symbols differentially expressed in contrast between both defined clusters and healthy controls was used as input. ImmQuant-provided DMAP dataset and pre-compiled lineage-tree was used as reference. Relative expression was computed respective to control data. Immune cell-type quantities were identified per subject. Median cell-type quantity values for cluster-assigned subjects were calculated. The resulting immune-related cell composition was compared between clusters.

*Model-generation for diagnosis and cluster stratification*

10 highly dysregulated genes were manually selected based on the biological rationale and earlier publications mentioning them: *IL1R2, CD177, HPGD, MMP8, HP, ARG1, OLFM4, HLA-DRB1, IL7R, AZU1*. The corresponding gene expression values of these genes from all 1,084 samples were imported to SPSS (V25, IBM Corporation, Armonk, USA). The dataset was randomly split in a 60:40 ratio into a derivation and validation dataset. Binary logistic regression analysis was performed using the derivation set with forward model building (inclusion: p<0.05, exclusion: p≥0.05; including constant term) to identify informative genes for both sepsis (both clusters) versus controls (diagnosis) respectively “Cluster 1” “Cluster 2” membership of patients with sepsis (stratification). Based on the resulting models (containing 5 genes for diagnosis and 6 genes for stratification), the corresponding probability for each sample to belong to the sepsis group respectively “Cluster 1” was computed. To evaluate the model performance regarding sensitivity and specificity, ROC-analysis was performed. For confirmation, the gene expression values of the validation set samples together with the regression coefficients reported from the derivation model were used to calculate the respective probability of these samples. Again, ROC analysis were performed on these data.

**References**

1. Huber W, Carey VJ, Gentleman R, Anders S, Carlson M, Carvalho BS, et al. Orchestrating high-throughput genomic analysis with Bioconductor. Nat. Methods. 2015;12:115–21.

2. Müller C, Schillert A, Röthemeier C, Trégouët D-A, Proust C, Binder H, et al. Removing Batch Effects from Longitudinal Gene Expression - Quantile Normalization Plus ComBat as Best Approach for Microarray Transcriptome Data. Kaderali L, editor. PLoS ONE. Public Library of Science; 2016;11:e0156594.

3. Gautier L, Cope L, Bolstad BM, Irizarry RA. affy--analysis of Affymetrix GeneChip data at the probe level. Bioinformatics. 2004;20:307–15.

4. Carvalho BS, Irizarry RA. A framework for oligonucleotide microarray preprocessing. Bioinformatics. 2010;26:2363–7.

5. Kuhn A, Luthi-Carter R, Delorenzi M. Cross-species and cross-platform gene expression studies with the Bioconductor-compliant R package 'annotationTools'. BMC Bioinformatics. BioMed Central; 2008;9:26.

6. Davis S, Meltzer PS. GEOquery: a bridge between the Gene Expression Omnibus (GEO) and BioConductor. Bioinformatics. 2007;23:1846–7.

7. Taminau J, Meganck S, Lazar C, Steenhoff D, Coletta A, Molter C, et al. Unlocking the potential of publicly available microarray data using inSilicoDb and inSilicoMerging R/Bioconductor packages. BMC Bioinformatics. BioMed Central; 2012;13:335.

8. Taminau J, Lazar C, Meganck S, Nowé A. Comparison of merging and meta-analysis as alternative approaches for integrative gene expression analysis. ISRN Bioinform. Hindawi Publishing Corporation; 2014;2014:345106–7.

9. Novianti PW, van der Tweel I, Jong VL, Roes KC, Eijkemans MJ. An Application of Sequential Meta-Analysis to Gene Expression Studies. Cancer Inform. 2015;14:1–10.

10. Hackstadt AJ, Hess AM. Filtering for increased power for microarray data analysis. BMC Bioinformatics. BioMed Central; 2009;10:11.

11. Le S, Josse J, Husson F. FactoMineR: An R package for multivariate analysis. Journal of Statistical Software. 2008;25:1–18.

12. Davenport EE, Burnham KL, Radhakrishnan J, Humburg P, Hutton P, Mills TC, et al. Genomic landscape of the individual host response and outcomes in sepsis: a prospective cohort study. Lancet Respir Med. 2016;4:259–71.

13. Ward JH. Hierarchical Grouping to Optimize an Objective Function. Journal of the American Statistical Association. 1963;58:236–&.

14. Ritchie ME, Phipson B, Wu D, Hu Y, Law CW, Shi W, et al. limma powers differential expression analyses for RNA-sequencing and microarray studies. Nucleic Acids Res. 2015;43:e47.

15. Huang DW, Sherman BT, Lempicki RA. Systematic and integrative analysis of large gene lists using DAVID bioinformatics resources. Nat Protoc. 2009;4:44–57.

16. Frishberg A, Brodt A, Steuerman Y, Gat-Viks I. ImmQuant: a user-friendly tool for inferring immune cell-type composition from gene-expression data. Bioinformatics. 2016;32:3842–3.
